# The first positive evidence that training improves triage decisions in Greece: evidence from emergency nurses at an Academic Tertiary Care Emergency Department

**DOI:** 10.1186/s12873-023-00827-5

**Published:** 2023-05-31

**Authors:** Sofia-Chrysovalantou Zagalioti, Barbara Fyntanidou, Aristomenis Exadaktylos, Konstantinos Lallas, Mairi Ziaka

**Affiliations:** 1 Department of Emergency Medicine, AHEPA University General Hospital, Aristotle University of Thessaloniki, 54636 Thessaloniki, Greece; 2 Department of Emergency Medicine, Inselspital, University Hospital, University of Bern, Bern, Switzerland; 3 Department of Oncology, School of Medicine, Faculty of Health Sciences, Papageorgiou General Hospital, Aristotle University, 56429 Thessaloniki, Greece; 4 Department of Internal Medicine, Thun General Hospital, Thun, Switzerland

**Keywords:** Triage, Triage training, Emergency nurses, Decision-making

## Abstract

**Background:**

Triage refers to the process of patient prioritisation in the emergency department (ED). This is based on the severity of the patient’s illness and is performed by emergency nurses (ENs). This has a pivotal role in ensuring patient safety and in ensuring that the ED operates smoothly – so continuous and accurate training are essential. As Emergency Nursing has been formally established in Greece since 2019, it is of the uppermost importance that all Greek ENs should be trained in the use of a standardised triage system. The present study aimed to evaluate the effect of triage training of ENs in the use of the Swiss Triage System (STS) after an intervention of one week.

**Methods:**

The effect of triage training was studied experimentally by comparing performance before and one week after training. A sample of thirty-six ENs from the University Department of Emergency Medicine at AHEPA University Hospital took part. The role of training in triage by the STS was assessed by completing the same self-administered questionnaire before and after a 45-minute e-learning program (presentation video of STS but with simulation scenarios) which was available during the period of a week. The post-training test was taken 2 weeks later, after the training process.

**Results:**

The most promising finding was that there was a significant improvement in the number of correct answers after the training in triage (*p<*0.001). A significant improvement was also detected (*p<*0.001) in the questions that tested vigilance in providing safe health services by ENs, whereas there was no significant association between the number of correct answers and years of emergency experience or level of education, - either before or after the intervention.

**Conclusions:**

Triage training seems to successfully improve effective and efficient triage. To the best of our knowledge, this is the first study that has demonstrated that triage training has a significant positive impact on triage performance by ENs in Greece. It is planned to support these findings by real time studies in an ED.

**Supplementary Information:**

The online version contains supplementary material available at 10.1186/s12873-023-00827-5.

## Background

Emergency and Critical Care Nursing exists as a recognized specialisation in the USA, Australia, Switzerland, England, and several other countries [1–4]. In Greece, it has been officially recognised as a specialisation since 2019 [5]. As this is an innovative scientific field in Greece, it is hoped that this will assure continuous improvement in patient care.

Triage plays a pivotal role in an Emergency Department (ED), as it categorises the urgency and severity of a medical situation and offers appropriate care management. It is therefore imperative to adopt an efficient triage system [6]. Triage is part of decision making in medicine, and correct and timely decision making brings the optimal result of appropriate management of how patients are treated and cared for. The ultimate result must be better care and a reduction in patient waiting times in the ED. It is important to study and identify an appropriate training program for the national health care system to which it is to be delivered. Each country has its specific characteristics that can influence the local program for triage training [7].

The use of triage manuals by nurses has been described in international publications. The most widely used programs include the U.S. Emergency Severity Index (ESI), the Canadian Triage and Acuity Scale (CTAS), the Australasian Triage Scale (ATS), the U.K. Manchester Triage System (MTS), and the Swiss Triage System (STS) [8]. In countries where these triage systems have been implemented, the nurses in the ED are trained in applying these programs [6]. Kotsiou O. et al. likened the situation of EDs in Greece to Aeolius and his bag of winds – in other words, chaos! It is therefore urgently necessary to proceed with a new approach to improve the situation [9].

The development and improvement of triage decision-making skills can be addressed through different educational strategies, supported by clinical experience, and this should serve to improve patient flow in the ED [10–12]. Simulation seems to be more effective than simple lectures in training nurses in triage [13, 14]. Nevertheless, a systematic review was held in Iran from 2010 to 2020 to identify the best method for teaching triage and this concluded that a mixed learning approach (workshop, lectures, simulation, and games) was the most effective triage training [15]. The main purpose is to involve as many nurses as possible. If we considered the shifts and the nurses’ daily life, a lecture with 24-hour access is to be preferred [16, 17].

No similar studies have been carried out in Greece. The initial step remains scheduled and qualified training of nurses in the ED. Besides training, many other factors influence the outcome of triage assessment, such as clinical experience and psychological factors [18, 19]. However, we can still intervene and modify staff training. This will help us to offer the optimal outcome to our patients.

Overcrowding in Greek EDs is a common problem. In countries where specialisation is already developed, it seems that training of nurses in triage improves the patient’s triage-management in the ED [15]. Bearing these points in mind, we have searched in vein through the medical literature and networks (in both English and Greek) to identify literature on the use and adoption of any triage system – with the emphasis on studies that evaluate an organised program for educational triage in Greece. The present study aims to evaluate the role of an educational triage program, based on the manual of the STS, in an Academic Tertiary Care Emergency Department.

## Methods

### Design

This was a pre- and post-test study in the same comparison emergency nurses (EN) group, and was designed to evaluate triage, by using the same questionnaire before and after the intervention of training.

The ENs of the University Department of Emergency Medicine at AHEPA University Hospital completed a questionnaire composed of forty closed-ended multiple choice questions covering various topics (demographics, cardiology, neurology, psychiatry, traumatology, pulmonology, gynaecology, gastroenterology, urology). Questions were designed using the patient triage manual -Schweizer Triage System_Version_1.10_June_2018 (STS) [20], which was translated into Greek. The questionnaire used in this study was developed for this study (Supplementary Material, Questionnaire). Its validity was confirmed by faculty members of Emergency Medicine in Switzerland (A.E. and M.Z.) who use this manual in their daily practice in the ED.

The training that followed lasted for 45-min via e-learning. The STS-manual was presented and analysed. The training covered the four-level emergency scale, the assessment of vital signs, and the categorization of the patient to corresponding triage category, depending on the cause of admission (disease), as well as the vital signs. Depending on the severity of the disease, patients were assigned to different urgency levels from 1 to 4, with 1 being the most urgent [21, 22]. Finally, six different simulation scenarios were examined, as developed from real triage cases (Table 1). The software contained pictures of STS, text, and a slideshow. Access to the e-learning material was free for a week, so that the nurses were able to log onto the presentation from work or at home at any time convenient to them.

**Table 1:** Triage vignettes and expected triage category

Vignette	Scenario description	Expected category
1	35-year-old male with a known history of lumbar disk herniation, presents with worsening pain since 7 hours without injury. Complaining of sphincter dysfunction, pain 7/10.	2
2	60-year-old male presents with chest pain after passing high-voltage electricity at work. Duration of contact seconds, no entry-exit point.	1
3	20 year-old male, tall and thin, presents with sudden onset of chest pain in the left and a feeling of not “filling the lungs with air”. Normal vital signs.	2
4	25-year-old female is evacuated with police escort due to reported violent behavior. During triage after calm and respectful dialogue, she calms down, however she remains relatively agitated. Unknown psychiatric history. She is safe for herself and people around her. Normal vital signs.	2
5	50 year-old male presents due to a bike accident, 8 hours previously - he was wearing a helmet. Without loss of consciousness- asymptomatic - with abrasions of the upper right extremity- he is receiving anticoagulants. Normal vital signs.	2
6	82 year-old female presents with haematochezia. Last major haematochezia during transport to the hospital.	1

The collected data were evaluated by sex, age, marital status, years of ED-experience, and level of education of the ENs. The population was divided into five age groups: age group A, above 16 and below 30 years; age group B, 31-40 years; age group C, 41-50 years; age group D, 51-60 years and age group E, over 60 years old. The marital status was defined as married, divorced, widowed, in a relationship and single. The primary endpoint of this study was to investigate whether the intervention group after the educational video would elicit higher triage competency than in the test with the same questionnaire before training. The secondary endpoints of our study was the correlation between triage competency and educational status and years of emergency experience. We also investigated whether the intervention group would be more vigilant than the control group in providing safe health services after the training video.

Within two weeks of the end of the training process, participants in the e-learning were re-evaluated with the same questionnaire and new data were collected.

### Participants and data collection

ENs of AHEPA University Hospital participated in this study from 7^th^ November to 21^st^ November 2022. Thirty-eight ENs were assigned to the survey group before the training. In total, thirty-six ENs attended the 45-minute video and responded to the same questionnaire after two weeks. The inclusion criteria were access to a computer that the EN could operate. Nursing students were excluded, as were nurses who failed to participate in one of the stages of the research process.

### Statistical analysis

Firstly, we conducted a descriptive analysis with mean and standard deviation for continuous, and frequencies for categorical variables. For normally distributed variables, Wilcoxon’s paired t test was used to compare the change in correct answers between pre- (Q1) and post-questionnaire (Q2). ANOVA and the Kruskal-Wallis test were used for variables with three or more categories. For categorical variables, the McNemar test was used to compare the change in the percent of correct answers after the training video. All statistical tests were two-sided and a *p* value <0.05 was considered significant.

Data analysis was conducted with IBM SPSS v28 and plots in Jamovi 2.2.5.

### Ethical approval

This study received approval from the AHEPA University Hospital (No. 4^th^/4.11.2022). All methods were performed in accordance with the relevant guidelines and regulations. All participants willingly engaged in the data collection and signed written informed consent forms. All the questionnaires were kept anonymous.

## Results

### Baseline characteristics

Thirty-eight ENs were assigned to the survey group before the training. ENs attended the 45-minute video and responded after two weeks to the same questionnaire. Two participants were excluded from the study due to missing data in Q2, leaving 36 participants for analysis. The highest proportion of participants was aged 41-50 (52.8%), and most of them (*n=*28, 77.8%) were women. Most participants were married (52.8%). Their educational background ranged from secondary education and vocational training (16.7%), to higher tertiary education (Technological Educational Institutes) (61.1%) to postgraduate education (MSc level) (22.2%). The emergency experience was defined in four levels: under 1 year of experience (2.8%), 1-2 years of nursing experience (33.3%), 2-5 years (16.7%) and five or more (47.2%) (Table 2).

**Table 2:** Baseline characteristics of participants

	N (%)
Gender	
Female	28 (77.8)
Male	8 (22.2)
Other	
Age	
<30	7 (19.4)
31-40	6 (16.7)
41-50	19 (52.8)
51-60	4 (11.1)
>60	
Family status	
Married	19 (52.8)
Divorced	4 (11.1)
Widowed	
In a relationship	4 (11.1)
Not in a relationship	9 (25)
Education level	
Secondary education and vocational training	6 (16.7)
Higher tertiary education	22 (61.1)
Postgraduate (MSc level)	8 (22.2)
Doctorates (PhD level)	
ED years	
Less than a year	1 (2.8)
1-2 years	12 (33.3)
2-5 years	6 (16.7)
>5 years	17 (47.2)

### Comparison of outcomes

**Fig. 1:**
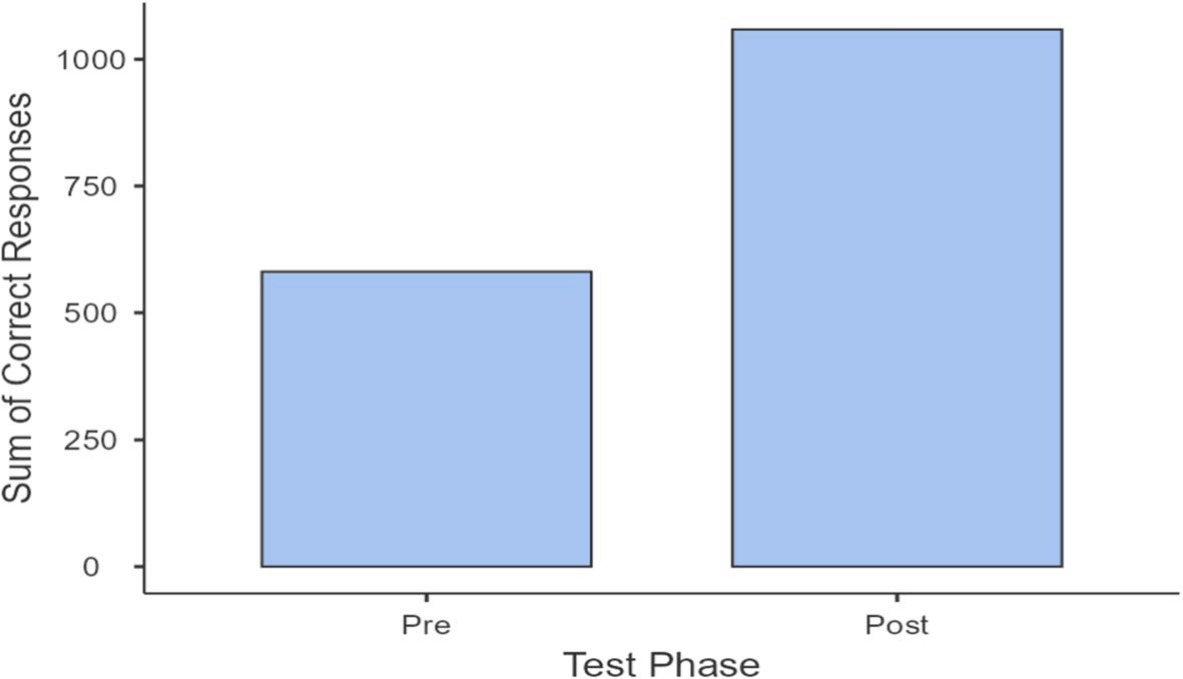
Summary of answers that were false at Q1 but correct at Q2

**Fig. 2:**
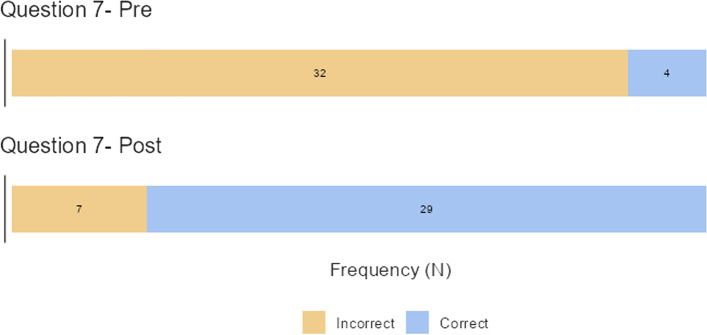
The question with the highest percent of change after the intervention

The first hypothesis of this study was that the intervention group after the training video would exhibit higher triage expertise when tested in the same follow-up questionnaire as the pre-training group. After the training intervention, the mean (range) change in the number of correct answers was 13.3 (4 - 20) and a statistically significant improvement in total correct answers was detected between Q1 and Q2 (McNemar χ^2^-test, *p<*0.001). After calculating the sum of correct questions, it was established that 500 answers that were false at Q1 were replaced by correct answers at Q2 (Fig. 1), but only 22 answers switched from right at Q1 to false at Q2. The greatest changes in the number of correct answers were found in question 7 (the symptoms associated with fever that classified the patient as triage 1) which decreased from 32/36 to 7/36, corresponding to a change of 69.5% (Fig. 2). Large decreases were also found in question 25 (that referred to triage classification of a patient with frostbites), which decreased from 19/36 to 6/36 (36.1% of change in incorrect responses).The smallest changes were found in question 8 (symptoms not associated with hypotension), where there was no change (*p=*0.999). Changes were also slight for question 24 (burn classification), for which the number of correct answers was 13/36 in Q1 and 16/36 in Q2, corresponding to 8.3% of change.

The second hypothesis of this study was the association either of the educational status or years of emergency experience with the triage expertise. Before the training session, the mean (SD) number of correct answers per participant was 16.1 (3.58) and there was no statistically significant difference for correct answers in Q1 in association with emergency experience (ANOVA Kruskal – Wallis, *p=*0.295) or with educational status (*p=*0.972). Likewise, for the change between Q1 and Q2, there was no significant association between the number of correct answers and either the years of emergency experience or the level of education (*p=*0.355 and 0.835, respectively).

**Fig. 3:**
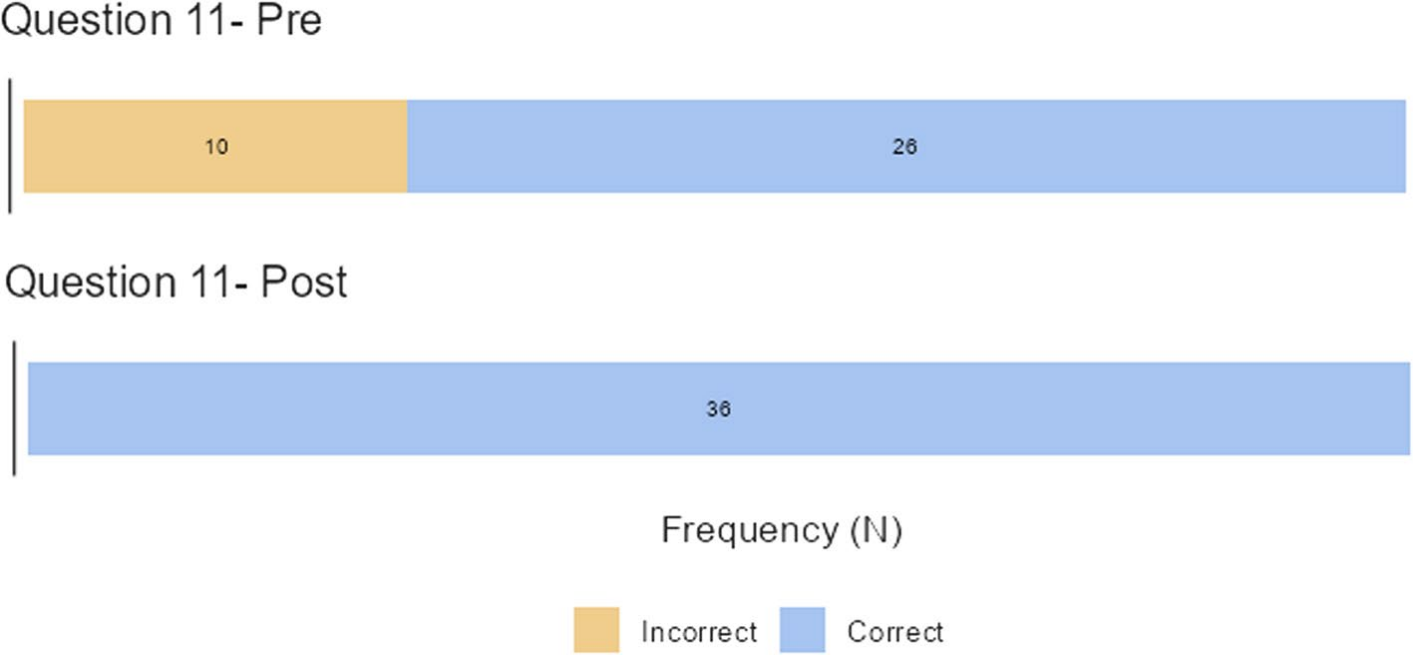
Question 11 tested the vigilance in providing safe health services

The third hypothesis of this study was that the intervention group would exhibit greater vigilance in providing safe health services after the training video than before. The questionnaire included two questions that tested the vigilance on providing safe health services, that is, Question 32 (the need of isolation in a 70-year-old-male presented with diarrhoea and discharged two days earlier after a pneumonia diagnosis) and Question 11 (what will be the first action in approaching a patient with headache, neck stiffness, fever, and purpuric rush?). For Question 32, there were 22 responses that switched from wrong in Q1 to correct in Q2 (66.7% and 5.6% incorrect responses, respectively; *p <* 0.001), while for Question 11, there were 10 responses that switched from wrong in Q1 to right in Q2 (27.8% and 0% incorrect responses, respectively, *p =* 0.002) (Fig. 3).

## Discussion

Multiple training programs seem to lead to a change in practice in triage [23]. These methods have also been widely investigated in testing the accuracy and evaluating the implementation of existing triage scales, such as ESI, CTAS and STS [24]. Any training procedure can be considered that aims to improve triage categorisation by ENs. Training approaches that improve practical outcomes include traditional learning with workshops, didactic lectures, case studies, simulation scenarios, and e-learning, accompanied by platforms for online discussion [25, 26]. Our hypothesis is that multimethod training will probably enhance performance.

The present study is the first recorded analysis of the implementation of triage training courses for ENs in Greece. We found a significant positive impact of triage training after using the STS manual. Similar study outcomes were concluded in an Iran study after using ESI triage in a two-day workshop and Canada after a 6 weeks online course in CTAS [17, 27]. The latter intervention also emphasised the effectiveness of an e-learning triage training program, an approach that is supported by many authors on training large groups, as this is a cost-effective and efficient method [15, 17, 26].

The current study showed that there was no association between educational status or years of emergency experience and the number of correct answers on triage competency. This is in contrast with the study of Considine J et al, where knowledge and educational status- seem to be more important than years of emergency nursing [28]. A possible explanation for the observed discrepancy could be that the majority of ENs in our study had at least two years experience in an ED. In addition, it is likely that the increased demands on emergency nursing care observed during the Corona Virus 19 (COVID-19) pandemic significantly enhanced the skills and competencies of nurses with less experience.

ENs are responsible for the safety of patients and others in the waiting room, and after implementation of our triage training, there were significant improvements. These results are not fully consistent with previous studies, who suggest that assessment of the overall triage process is required, rather than just answers to specific questions, in order to improve safety. [7, 29].

Nevertheless, the remote training techniques via web learning during COVID-19 greatly extend the scope of training, as this approach magnifies the possibility of participation - regardless of limiting factors, such as the obligation to be physically present at a specific place and time [30]. Literature suggests that factors such as educational status may help to achieve the training goals and to encourage more nurses to take part in this educational triage process [25]. Therefore it would be interesting to include questions assessing the willingness to participate in the training and the satisfaction of the trainees after its completion, which was not contained in the present study. E-learning technologies are a flexible way to maximise the benefits of education in ED triage. Delivering web courses leads to drastic changes in emergency nursing [17, 31, 32]. Effective computer-based education is vital for emergency nursing due to the nature of shift work, where traditional face-to-face teaching may be difficult. Current studies indicate that significant improvements in triage outcomes may be achieved by interactive web blended learning, including lectures, photos, videos, scenarios [12, 33].

Similar research aims to identify a well based training framework to improve the accuracy of decisions by ED nurses in the prioritisation of patient care [34]. Proper triage enhance patients’ safety. Mistriage is divided into undertriage, which refers to the underestimation of the patient’s severity, and overtriage, which refers to the overestimation of the urgency of a condition. The results of triage errors have a negative impact on patient safety, on quality of provided healthcare and cose extra cost in hospital logistics. Assessment of over- and under-triage before and after using a triage scale has been shown to increase the accuracy of patient categorisation after training in using a triage scale [35, 36].

Despite the strengths of the present study, some limitations should be taken into consideration. Specifically, the present study was a single centre study. Moreover, the sample size and the duration of the study were small. Perhaps a study with more trainees and a longer training program would have given different results. Such studies are restricted by personal limitations such as the computer and Internet skills of the participants, as well as more general limitations of Greek legislation. As a new field, Emergency Nursing has to overcome the chronic barriers of a health system that has only recently included Emergency Medicine. Hence there is no established legislation on triage implementation by nurses and no effective triage system to be applied in Greek hospitals.

## Conclusions

Triage plays a crucial role in an ED, as it promotes patient safety and effective decision-making in the prioritisation of immediate and critical medical situations, thereby improving patient care. It is therefore necessary to amend the current legislation, in order to define an appropriate and effective triage system, which will significantly improve the use of the available hospital resources. This implementation depends on and requires the training of ENs to achieve correct triage and optimal efficiency of this system. However, the data on optimal training of ENs are limited, especially in Greece. The present study has tried to fill this gap by showing that triage training of ENs by using e-learning with presentation and simulation scenarios has a positive impact. Although further research is required on how this new approach would benefit the overcrowded ED and patients’ waiting times, this is the first report on effective triage training within the Greek health system. The findings will be applied in practice.

## Supplementary Information

**Additional file 1.**

## Data Availability

The datasets used and/or analysed during the current study are available from the corresponding author on reasonable request.

## Abbreviations

ED: Emergency Department
EN: Emergency Nurse
ESI U.S.: Emergency Severity Index
CTAS: Canadian Triage and Acuity Scale
ATS: Australasian Triage Scale
MTS U.K.: Manchester Triage System
STS: Swiss Triage System (STS)
Q1: pre-questionnaire
Q2: post-questionnaire
