# Supplementary material for: The first positive evidence that training improves triage decisions in Greece: evidence from emergency nurses at an Academic Tertiary Care Emergency Department
Source: BMC Emerg Med. 2023 May 31;23:60. doi: 10.1186/s12873-023-00827-5 (PMC10229392; doi:10.1186/s12873-023-00827-5)
Supplement: Supplementary file 1 — Additional file 1. [file 12873_2023_827_MOESM1_ESM.docx]

**QUESTIONNAIRE**

**1.** Gender

a) Male

b) Female

c) None of the above

**2.** Age

a) <30

b) 31-40

c) 41-50

d) 51-60

e) >60

**3.** Marital status

a) Married

b) Divorced

c) Widowed

d) In a relationship

e) Not in a relationship

**4.** Level of education

a) secondary education and vocational training

b) higher tertiary education (Technological Educational Institutes)

c) postgraduate education (MSc level)

d) Doctorates (PhD level)

**5.** How many years have you been working in the ED?

a) Less than a year

b) 1-2 years

c) 2-5 years

d) >5 years

**6.** Tachycardia. Which of the following is suggested?

a) measure pulse manually

b) measure pulse with an oximeter

c) measure temperature

d) check if it is rhythmic or irregular

e) b and d

f) all of the above

**7.** In a patient with a fever. Which of the following accompanying symptoms will result in the patient being classified as Triage 1?

a) impaired general condition

b) history of chemotherapy

c) photophobia

d) petechiae

**8.** Which of the following symptoms is not associated with hypotension?

a) dizziness

b) weakness

c) chest pain

d) loss of consciousness

**9.** Patient presents stressed with BP of 200mmHg, without chest pain or other associated symptoms. He is classified as Triage 2.

a) correct

b) wrong

**10.** Which of the following symptoms suggests a venous etiology of lower extremity edema and pain?

a) limb pallor

b) no pulse in foot

c) mobility and sensation disorder

d) bluish-purple discoloration of the skin

**11.** Patient with headache, neck stiffness, fever and purpuric rash. What would be your first action upon approaching the patient?

a) assess vital signs

b) use of protective and safety equipment (e.g. mask)

c) keeping the patient in a common waiting area

d) triage to category 2 and await dermatological assessment

**12.** A 18-year-old patient presents with dyspnea, tachypnea and abdominal pain. Which of the following is necessary to assess to categorize him in Triage?

a) BP

b) SpO2

c) blood sugar test

d) all of the above

e) b and c

**13.** Which of the following is not associated with acute confusional state ?

a) drug use

b) infection

c) mental illness

d) urinary retention

e) none of the above

**14.** A patient with before fainting symptoms. When is he **NOT** classified in the Triage 2 category?

a) appearance in a supine position

b) after changing from a sitting to a standing position

c) with accompanying chest pain

d) occurrence during fatigue

**15.** A patient with a known psychiatric history presents with agitation accompanied by intense anxiety, but no suicidal ideation. What triage category is he in?

a) 1

b) 2

c) 3

d) 4

**16.** Which score helps to categorize patients with alcohol withdrawal into Triage category 2 or category 3?

a) CIWA

b) COWS

c) both

d) none of the above

**17.** Which score helps to categorize opioid withdrawal patients into Triage category 2 or category 3?

a) CIWA

b) COWS

c) both

d) none of the above

**18.** In which case Triage is overlooked?

a) polytrauma patient

b) loss of a limb

c) spinal injury with neurological impairment

d) none of the above

**19.** When do you classify an injured person with chest trauma as Triage 1?

a) involvement in a car accident with the passenger dead

b) fall from a height of more than 6 meters

c) history of hemophilia or taking anticoagulants

d) open chest wound

**20.** Patient with pain below the left hemithorax reports a car accident three days ago. What triage category is he classified in?

a) 1-2

b) 2-3

c) 3-4

**21.** Patient ambulatory after a car accident with nosebleed.

a) belongs to Triage 1-2 category

b) waiting to be examined by an ENT

c) waiting to be examined by a surgeon

d) waiting to be examined by a neurologist

**22.** Patient with traumatic brain injury. When is he classified as Triage 1?

a) with accompanying nausea-vomiting

b) taking anticoagulants/ hemophilia history

c) with neurological semiology

d) with ear bleeding/ rhinorrhea

**23.** When is a patient with a deep injury classified as Triage 1? When it is identified:

a) in the chest

b) in the abdomen

c) in the perineum

d) all of the above

e) in (a) and (b)

**24.** A burn victim with 2nd and 3rd degree burns and distribution only to the trunk is classified as Triage 2 when the percentage of distribution is:

a) <10%

b) 10-20%

c) 20-30%

d) >30%

**25.** A patient with frostbite classified in the Triage category:

a) 1-2

b) 2-3

c) 3-4

**26.** A patient with animal vomit is assigned to Triage 2 when:

a) when it is a poisonous insect or animal

b) there is a nerve-vascular injury

c) suspected compartment syndrome

d) in all of the above

e) b) and c)

**27.** Patient with melena voiding reports a large loss at home and a voiding on admission. He is taking anticoagulants for atrial fibrillation. Classified as a triage category:

a) 1

b) 2

c) 3

d) 4

**28.** A patient with abdominal pain. When is he classified as Triage 2?

a) with accompanying fever

b) acute pain described as a stabbing pain

c) with inability to pass stool and gas

d) with reported use of anticoagulants

e) all of the above

f) (a), (b) and (c)

**29.** A patient presents with vomiting in which possible triage categories is he classified in?

a) 2 or 3

b) 3 or 4

c) 2 or 3 or 4

**30.** A patient with vomiting before 5 hours. Which triage category is he classified in?

a) 2

b) 3

c) 4

**31.** 40-year-old female with epigastric pain and vomiting. Which questions should be asked during triage categorization?

a) history of coronary artery disease

b) possibility of pregnancy

c) use of medication

d) all of the above

e) a) and c)

**32.** A 70-year-old patient was hospitalized for pneumonia and discharged two days ago. She is hospitalized due to diarrhea.

a) she is assigned to Triage and awaits his turn

b) has priority due to age and the high likelihood of dehydration

c) needs isolation

d) none of the above

**33.** A 65-year-old female patient with hypogastric pain is taking anticoagulants. In which triage category is she classified?

a) 1

b) 2

c) 3

d) 4

**34.** A 28-year-old pregnant patient is classified in Triage 1 when:

a) the child has already been born

b) she arrives with contractions lasting > 2min

c) she comes with contractions lasting < 2min

d) a) and b)

e) a) and c)

**35.** A pregnant woman comes forward stating that she "does not feel the movements of the fetus". Which triage category is she in?

a) 1

b) 2

c) 3

d) depending on the vital signs of the mother and fetus

**36.** A patient with macroscopic hematuria who is not receiving any other medication. To which triage category is he assigned?

a) 1

b) 2

c) 3

d) 4

**37.** A patient with priapism in which triage category is he classified?

a) 1

b) 2

c) 3

d) 4

**38.** A female patient with chronic vaginal prolapse, urinary incontinence since three days and an impaired general condition in which triage category is she classified?

a) 1

b) 2

c) 3

d) 4

**39.** A female patient with cancer who is undergoing chemotherapy and immunotherapy, presents with fever up to 38.5°C. In which triage category is she classified?

a) 1

b) 2

c) 3

d) 4

**40.** A patient with hypothermia faces the potential risk of arrhythmias; does the patient need special handling by health care personnel?

a) Correct

b) False
